# A Medical Causerie

**Published:** 1915-04-17

**Authors:** 


### A MEDICAL CAUSERIE.

The election of a new President of the Royal College of Physicians is an event which hardly attracts much interest or even attention in the general body of the profession. Still, the office is a very interesting one, and is dignified by many proud associations and worthy traditions. Among the staffs of the hospitals from which the President is usually taken there is naturally much ambition to secure the election, and stories of diplomatic manœuvring as an aid to success are not unknown. The official return may suggest a unanimous election, but this conclusion is by no means always correct. Indeed, the contest is often acute and the majority one of small proportions. The College, like many other ancient corporations, has lost much of its power, but it continues to be looked to as an authority on the standard of professional conduct. In some sense the President is perhaps the leader of the profession, and he is sure of the offer of a title and of an unfailing flow of invitations to City dinners and other official functions.

The influenza scourge continues to be very active in London and the neighbourhood, and many general practitioners are consequently overwhelmed with work. For the most part the cases are mild in type and suggest some form of epidemic catarrh rather than genuine influenza. Still, there is a proportion of instances in which serious respiratory complications ensue, and this development is displayed in the increased death-rate from these causes. The latest figures show almost twice as many deaths from pneumonia, bronchitis, and the like as were recorded in the corresponding period of 1914.

Sir Alfred Keogh's explanation of the wishes of the military medical authorities has helped to clear the air. Evidently it has been recognised that unless by part-time service it is impossible to meet the conditions created by the war. The younger men may accept commissions in the R.A.M.C., and may thus place themselves entirely at the disposal of the War Office; but the seniors, with few exceptions, have engagements to the civil population which it is impossible to ignore. Many, however, are more than willing to give steady and systematic help, provided the conditions are not inconsistent with other duties which are equally necessary for the care of the community. Now that this has been recognised at headquarters it rests with the profession to develop a scheme which will satisfy the official conditions and will at the same time secure due attention to our civil hospitals and to general practice. There is one rather delicate matter which the hospital staffs and governing bodies will have to face—namely, the propriety of continuing to invite young practitioners to accept positions as house physicians and house surgeons. How can this fairly be continued in view of the official statement that the want of young doctors for whole-time service with the Army is a national emergency? Men who are physically unfit can remain, but surely others are bound to answer the country's call. If women residents cannot be obtained it may be fairly argued that it rests with the visiting staff either to organise the services of neighbouring practitioners, or themselves to undertake in turn the usual duties of the resident staff. To keep healthy, vigorous young men at home doing work which can be performed by others is a proceeding which in such a time as the present is hardly capable of defence.

A good deal of criticism is being heard in reference to the position of the medical officers on the staffs of the general military hospitals. These gentlemen hold what are termed *à la suite* commissions—that is, in time of peace they have neither pay nor duties, but on mobilisation they assume their military titles and receive the pay of their rank. Their duties attach them to the care of the sick and wounded in the local military hospitals, but it appears that they are allowed also to continue their private practices. This arrangement necessarily means that the staffs of these hospitals must be much larger than were whole-time officers employed, and consequently the expense to the country is much greater. It would be interesting to have a record of the exact amount of time given to military service by gentlemen who occupy these positions. The question is all the more acute seeing that many members of the Territorial R.A.M.C., who even in peace have given time and attention to the country's defence, are now called away from their practices for service with the military units to which they belong. It therefore comes to this, that those who have made sacrifices during peace have to make greater ones during war, whilst those who did nothing prior to the war now draw their pay under conditions which allow them still to earn their usual incomes.

The proposal to institute a "Forget-Me-Not Day" on which ladies were to sell flowers in the streets of London for the benefit of soldiers maimed in the war has suffered a series of shocks. The public meeting at the Savoy Hotel attracted only a scanty audience, and it heard some very vigorous criticism from Lord Knutsford, who protested against further street collections. "Rose Day" is allowable, as it is unique, but a multiplication of such days would vulgarise the whole series. The speaker thought little credit was due to those who practised piracy in the world of ideas, and pleaded for "honesty among beggars." All this attracted little support in the meeting, but its effect seems to have been considerable, as some of the leading ladies have suddenly discovered urgent private affairs which make it impossible for them any longer to continue to hold office. Discarding the touching appeal of the flower which they professed, they seem anxious now to be quickly and completely out of mind. It is stated that the "day" is postponed until September, and this leaves abundant time for still further resignations.

